# Underlying Cause and Place of Death Among Patients With Amyotrophic Lateral Sclerosis in Taiwan: A Population-Based Study, 2003–2008

**DOI:** 10.2188/jea.JE20130045

**Published:** 2013-11-05

**Authors:** Ching-Piao Tsai, Bo-Hsiung Chang, Charles Tzu-Chi Lee

**Affiliations:** 1 Neurology, Neurological Institute, Taipei Veterans General Hospital and National Yang-Ming University, Taipei, Taiwan; 2 Department of Public Health, Kaohsiung Medical University, Kaohsiung, Taiwan

**Keywords:** amyotrophic lateral sclerosis, cause of death, respiratory disease, suicide

## Abstract

**Background:**

Few studies have assessed cause of death among patients with amyotrophic lateral sclerosis (ALS). We investigated underlying cause and place of death among patients with ALS in Taiwan during 2003–2008.

**Methods:**

The data source was the Taiwan National Health Insurance database for the period 2003–2008. In total, 751 patients older than 15 years with a primary diagnosis of ALS were included and followed until 2008 in the national mortality database. Crude mortality rates (per 100 person-years) and standardized mortality ratios (SMRs) were calculated in relation to cause of death, sex, and age group (15–44, 45–64, 65+ years).

**Results:**

In total, 297 (39.6%) patients died during the follow-up period, an age- and sex-standardized mortality rate 13 times (95% CI, 10.6–15.6) that of the Taiwanese general population. The leading cause of death among the patients was respiratory diseases, and the second most frequent cause was cardiovascular diseases. During the first year after an ALS diagnosis, suicide was much more frequent (SMR, 6.9; 95% CI, 1.9–17.6) than among the general population.

**Conclusions:**

During 2003–2008, respiratory diseases and cardiovascular diseases were the most frequent causes of death among Taiwanese patients with ALS. In addition, our findings indicate that suicide prevention is an urgent priority during the period soon after an ALS diagnosis.

## INTRODUCTION

Amyotrophic lateral sclerosis (ALS) is a fatal neurodegenerative disease characterized by a progressive loss of motor neurons in the spinal cord.^1^ ALS is not itself fatal, so deaths among patients with ALS are caused by other diseases. Although treatment with riluzole and tracheotomy can extend survival,^2^^,^^3^ most patients with ALS die from respiratory failure within 2 to 5 years.^4^ The prevalence and incidence of ALS are reported to be increasing in Hong Kong,^5^ Japan,^6^ and Sweden,^7^ and may be increasing worldwide; however, there is limited information on cause of death and mortality rates in relation to disease category among patients with ALS.^1^^,^^8^^,^^9^

An improved understanding of causes of death among patients with ALS might improve end-of-life palliative care. In addition, determining the place of death (ie, inside or outside a medical facility) could help identify a need for highly specialized care, eg, hospice and nursing home care. Therefore, we investigated the cause and place of death among Taiwanese patients with ALS, using data for the period 2003–2008.

## METHODS

### National health insurance in Taiwan

The Taiwan National Health Insurance (NHI) program is a government-run single-payer insurance system that was established in 1995. By December 2010, there were 23.074 million individuals enrolled in the program nationwide, with a coverage rate of 99.6%. Registration of all cases of serious disabling diseases (SDD), such as chronic renal failure, myasthenia gravis, cancer, and ALS, is required by the NHI bureau before SDD certification can be granted. Using the El Escorial criteria,^10^ a group of neurology specialists at the Taiwan Bureau of National Health Insurance (BNHI) reviewed the medical records of patients with ALS. There were 37 099 registered medical doctors and 553 neurology specialists in Taiwan in 2008, and 790 621 patients with SDD certification—3.4% of the national population.

### Sample

This was a retrospective population-based study. The research data were obtained from the National Health Insurance Research Database (NHIRD) provided by the National Health Research Institute, which included information on outpatient care, ambulatory care, inpatient care, and dental services. ALS cases were identified by using code 335.20 from the International Classification of Diseases, Ninth Revision (ICD-9). The study data comprised information from all medical claims by ALS patients during the period from January 1, 2003 through December 31, 2008. Only ALS patients with SDD certification who were older than 15 years at diagnosis were included. Patients with SDD certificates are eligible for exemption from insurance premiums and copayments, and approval of SDD certificates requires strict evaluation by the Taiwan Department of Health, Executive Yuan. In this study, all ALS cases were verified by linking encrypted identification numbers with SDD certificates. All investigated ALS cases had SDD certificates and were followed until December 31, 2008, using the national mortality database. The approval of an institutional review board was not necessary, because the data provided by the NHIRD lacked identifying information.

### Classification of underlying cause of death

We classified cause of death by disease group: (1) respiratory diseases, (2) cardiovascular diseases, (3) metabolic diseases, (4) diseases of the urinary system, (5) cancer, (6) suicide, (7) diseases of the digestive system, (8) diseases of the musculoskeletal system or connective tissue, (9) symptoms, signs, and ill-defined conditions, and (10) diseases of the nervous system (Table 1).

**Table 1.** Cause of death classification used in the present study of patients with amyotrophic lateral sclerosis

Cause of death	ICD-9 code
Respiratory diseases	460–519
Cardiovascular diseases	390–459
Metabolic diseases	240–279
Urinary system diseases	580–629
Cancer	140–239
Suicide	E950–959, E980–989
Digestive system diseases	520–578
Diseases of the musculoskeletal system or connective tissue	710–739
Symptoms, signs, and ill-defined conditions	780–799
Nervous system diseases	320–339, 341–359

ICD-9: International Classification of Diseases, Ninth Revision.

### Statistical analysis

Age- and sex-specific mortality rates from Taiwanese mortality statistics from January 1, 2003 through December 31, 2008 were used to calculate expected number of deaths. Standardized mortality ratios (SMRs) within 1 year were calculated as the ratio of observed to expected numbers of deaths. SMRs and crude mortality rates (per 100 person-years) were calculated in relation to cause of death, sex, and age group (15–44, 45–64, 65+ years). The crude mortality rate during the overall follow-up period was calculated by the “person-years-at-risk” method. All cases were traced from the date of first ALS diagnosis until December 2008. Calculations were performed using SAS version 9.2.

## RESULTS

Among a total of 751 ALS patients, 297 (39.5%) died during the follow-up period. Mean (SD) follow-up time was 2.3 (1.5) years. Annual incidence was 0.55 (per 100 000) in Taiwan during 2003–2008. The annual crude mortality rate for ALS was 17.6 (per 100 person-years). Among the 297 patients with ALS who died, 194 were male and 103 were female (male-to-female ratio 1.88). Most deaths were due to respiratory diseases (*n* = 151, mortality rate = 8.9 per 100 person-years; Table 2).

**Table 2.** Mortality rate by cause of death and sex among patients with amyotrophic lateral sclerosis in Taiwan, 2003–2008

Underlying cause of death	Males		Females		All	
		
	*n*	Mortality^a^	*n*	Mortality^a^	*n*	Mortality^a^
Respiratory diseases	98	9.14	53	8.58	151	8.93
Cardiovascular diseases	17	1.58	15	2.43	32	1.89
Metabolic diseases	9	0.84	5	0.81	14	0.83
Urinary system diseases	8	0.75	3	0.49	11	0.65
Cancer	8	0.75	1	0.16	9	0.53
Suicide	5	0.47	1	0.16	6	0.36
Digestive system diseases	5	0.47	0	0	5	0.30
Diseases of the musculoskeletal system or connective tissue	8	0.75	2	0.32	10	0.59
Symptoms, signs, and ill-defined conditions	27	2.52	16	2.59	43	2.54
Nervous system diseases	3	0.28	2	0.32	5	0.30
Unknown	6	0.36	5	0.81	11	0.65

Total	194	17.99	103	16.84	297	17.57

^a^Per 100 person-years.

Thirty-one (10.4%) patients died before age 45 years, 121 (40.7%) died at age 45 to 64 years, and 145 (48.8%) died at age 65 years or older. Cardiovascular diseases, metabolic diseases, diseases of the urinary system, and cancer were frequent causes of death among patients aged 65 years or older at death. Death due to diseases of the digestive system was the second most frequent cause of death among patients younger than 45 years. Death due to respiratory diseases was more frequent among patients aged 45 to 64 years and those aged 65 years or older than among younger patients (Table 3).

**Table 3.** Age at death by cause of death among patients with amyotrophic lateral sclerosis in Taiwan, 2003–2008

Underlying cause of death	Age at death						All	

	15–44		45–64		≥65			
			
	*n*	Mortality^a^	*n*	Mortality^a^	*n*	Mortality^a^	*n*	Mortality^a^
Respiratory diseases	13	2.93	71	8.65	67	15.71	151	8.93
Cardiovascular diseases	2	0.45	9	1.10	21	4.93	32	1.89
Metabolic diseases	1	0.23	5	0.61	8	1.88	14	0.83
Urinary system diseases	0	0	3	0.37	8	1.88	11	0.65
Cancer	0	0	1	0.12	8	1.88	9	0.53
Suicide	1	0.23	4	0.49	1	0.23	6	0.36
Digestive system diseases	3	0.68	1	0.12	1	0.23	5	0.30
Diseases of the musculoskeletal system or connective tissue	1	0.23	5	0.61	4	0.94	10	0.59
Symptoms, signs, and ill-defined conditions	8	1.81	16	1.95	19	4.46	43	2.54
Nervous system diseases	1	0.23	2	0.24	2	0.47	5	0.30
Unknown	1	0.23	4	0.49	6	1.23	11	0.65

Total	31	7.00	121	14.75	145	34.01	297	17.57

^a^Per 100 person-years.

Among the 297 deaths, 154 (53.5%) occurred at a medical facility and 134 (46.5%) occurred outside a medical facility. All 6 cases of suicide occurred outside a medical facility. Two-thirds of deaths due to respiratory diseases occurred in a medical facility. There were missing data on place of death for 9 patients (Table 4).

**Table 4.** Place of death by cause of death among patients with amyotrophic lateral sclerosis in Taiwan, 2003–2008

Cause of death	Inside medical facility		Outside medical facility	
	
	*n*	%	*n*	%
Respiratory diseases	100	66.7	50	33.3
Cardiovascular diseases	14	45.2	17	54.8
Metabolic diseases	6	46.2	7	53.8
Urinary system diseases	8	72.7	3	27.3
Cancer	4	44.4	5	55.6
Suicide	0	0.0	6	100.0
Digestive system diseases	1	20.0	4	80.0
Diseases of the musculoskeletal system or connective tissue	4	40.0	6	60.0
Symptoms, signs, and ill-defined conditions	16	38.1	26	61.9
Nervous system diseases	1	25.0	3	75.0
Unknown	0	0.0	7	100.0

Total	154	53.5	134	46.5

Data on place of death were missing for 9 patients.

The SMR for all-cause death was 12.9 (95% CI, 10.6–15.6); the SMR for females (21.5; 95% CI, 15.3–29.4) was significantly higher than that for males (9.5; 95% CI, 7.4–12.0). All causes of death were more prevalent among patients with ALS than among the general population, except for cancer, diseases of the urinary system, and diseases of the digestive system. The SMR was 49.6 (95% CI, 35.9–66.8) for respiratory diseases, 11.7 (95% CI, 7.2–17.8) for cardiovascular diseases, and 6.9 (95% CI, 1.9–17.6) for suicide (Table 5).

**Table 5.** Standardized mortality ratios by cause of death and sex among patients with amyotrophic lateral sclerosis in Taiwan, 2003–2007

Cause of death	Males	Females	All
Respiratory diseases	29.2 (19.1–42.8)	131.8 (76.8–211.1)	49.6 (35.9–66.8)
Cardiovascular diseases	6.4 (3.1–11.7)	29.2 (14.5–52.2)	11.7 (7.2–17.8)
Metabolic diseases	5.3 (1.4–13.5)	4.5 (1.2–12.5)	5.1 (1.3–13.4)
Urinary system diseases	1.2 (0.3–3.3)	—^a^	1.2 (0.3–3.3)
Cancer	1.1 (0.2–3.2)	—^a^	1.1 (0.2–3.2)
Suicide	5.6 (1.1–16.3)	8.8 (0.1–48.9)	6.9 (1.9–17.6)
Digestive system diseases	1.6 (0.0–9.0)	1.5 (0.0–8.5)	1.6 (0.0–9.0)
Diseases of the musculoskeletal system or connective tissue	182.5 (58.8–425.9)	103.3 (1.4–574.9)	164.1 (59.9–357.1)
Symptoms, signs, and ill-defined conditions	14.8 (5.4–32.4)	24.7 (2.8–89.0)	17.8 (7.6–35.0)
Nervous system diseases	14.2 (0.2–79.2)	51.0 (0.7–283.8)	23.8 (2.7–85.9)

All causes of death	9.5 (7.4–12.0)	21.5 (15.3–29.4)	12.9 (10.6–15.6)

^a^Could not be calculated due to small sample size.

## DISCUSSION

This is the first study to investigate cause of death among patients with ALS in Taiwan. There were 11 (3.7%) patients with missing data on underlying cause of death, and 43 (14.5%) patients for whom cause of death was coded as symptoms, signs, and ill-defined conditions. Therefore, the mortality rates for specific underlying diseases were underestimated in this population-based study. However, our finding of markedly higher mortality rates for certain disease categories among patients with ALS as compared with rates in the general population would not be substantially altered by the presence of such underestimation. In addition, any underestimation of suicide mortality is likely to be limited, due to the great attention that suicide cases receive in the Taiwan national mortality database. The proportion of patients with an undetermined cause of death in the present study was consistent with previously reported values; 1 such study reported that cause of death was unknown in 13% of ALS cases, even though information on mortality was obtained directly from the patients’ neurologists.^11^ In addition, the clinical condition of patients, and thus cause of death, varies with the severity of ALS. Unfortunately, we lacked information on ALS severity.

Respiratory diseases was the most frequent cause of death (151 of 297 deaths, 50.8%), which is consistent with the findings of previous reports.^1^^,^^8^^,^^11^ However, the proportion of deaths due to respiratory diseases in this study (50.8%) was much lower than in Italy (81.3%),^1^ France (80%),^11^ and southwest China (65.5%).^8^ The second most frequent cause of death was cardiovascular diseases in this study, but such deaths were infrequent in Italy.^1^ The second most frequent causes of death were sudden death and death during sleep, in the Italian study,^1^ and nutrition-related disorders, in the study conducted in southwest China.^8^ Most of the present patients died in a medical facility, but most patients died at home in the studies of patients with ALS in southwest China^8^ and Italy.^1^ These disparities are likely due to economic and cultural differences. In addition, because all Taiwanese patients with ALS receive highly subsidized medical care through the National Health Insurance System, they might have received a higher standard of care.

As compared with rates among the Taiwanese general population, most causes of death were more frequent among patients with ALS, except cancer, diseases of the urinary system, and diseases of the digestive system. The absence of a difference in the mortality rates for these diseases may due to the small numbers of patients who died from these causes and the resulting limited statistical power. In addition, the absence of a significant difference in the cancer mortality rate is likely due to the younger age at death among patients with ALS. A previous study in Taiwan found that the mean (SD) age of patients at ALS diagnosis was 56.27 (14.15) years and that mean survival time was 5.6 years.^2^ In our data, 8 of the 9 cancer deaths were among patients older than 65 years. However, most ALS patients died before age 65 years, which reduced the age-related risk of cancer death.

As compared with women, men had a similar crude mortality rate but a lower SMR. This is primarily due to the known difference in mortality between men and women (ie, the mortality rate is higher among adult men in the general population).

A population-based cohort study in Sweden (which does not have legalized euthanasia or assisted suicide) investigated suicide risk among patients with ALS during 1965–2004. They reported a 6-fold increase in suicide risk among ALS patients (SMR 5.8; 95% CI, 3.6–8.8). The highest relative risk for suicide was observed during the first year after the first hospitalization.^12^ Euthanasia and assisted suicide are illegal in Taiwan. In our study, the age- and sex-standardized suicide rate after an ALS diagnosis was 7 times that of the general population, and, in our data, all 6 deaths due to suicide occurred during the first 6 months after an ALS diagnosis (range, 68–186 days). The risk of suicide was higher during the earlier phase of ALS, which was also the case in a study by Fang et al,^12^ who hypothesized that (1) patients experienced severe emotional strain while waiting for the diagnosis to be confirmed and (2) patients must be physically capable of performing suicide. Among the present 6 cases of suicide (5 men and 1 woman), mean age at death was 58.3 years (range, 51.0–63.6 years), which was lower than that among patients who died of other causes (*P* < 0.001). This result supports the notion that psychopathologic diagnosis is increasingly important in ALS.^13^ It has been suggested that men respond more strongly to poor economic conditions. Middle-aged men in Taiwan are culturally expected to support their family financially.^14^ Therefore, ALS may have a more devastating impact on middle-aged men in Taiwan, potentially leading to suicide, as suggested by our findings. Among Taiwanese, suicide prevention should be an urgent priority in the early period after an ALS diagnosis, especially among middle-aged men.
